# Aptamer-Based Technologies in Foodborne Pathogen Detection

**DOI:** 10.3389/fmicb.2016.01426

**Published:** 2016-09-12

**Authors:** Jun Teng, Fang Yuan, Yingwang Ye, Lei Zheng, Li Yao, Feng Xue, Wei Chen, Baoguang Li

**Affiliations:** ^1^College of Food Science and Engineering, Hefei University of Technology, HefeiChina; ^2^Animal Quarantine Laboratory, Jiangsu Entry-Exit Inspection and Quarantine Bureau, NanjingChina; ^3^Center for Food Safety and Applied Nutrition, U.S. Food and Drug Administration, Laurel, MDUSA

**Keywords:** aptamers, SELEX, ligands, aptamer-based biosensors, bacterial pathogen detection, dissociation constants, biomolecular screening, high affinity

## Abstract

Aptamers are single stranded DNA or RNA ligands, which can be selected by a method called systematic evolution of ligands by exponential enrichment (SELEX); and they can specifically recognize and bind to their targets. These unique characteristics of aptamers offer great potentials in applications such as pathogen detection and biomolecular screening. Pathogen detection is the critical means in detecting and identifying the problems related to public health and food safety; and only the rapid, sensitive and efficient detection technologies can enable the users to make the accurate assessments on the risks of infections (humans and animals) or contaminations (foods and other commodities) caused by various pathogens. This article reviews the development in the field of the aptamer-based approaches for pathogen detection, including whole-cell SELEX and Genomic SELEX. Nowadays, a variety of aptamer-based biosensors have been developed for pathogen detection. Thus, in this review, we also cover the development in aptamer-based biosensors including optical biosensors for multiple pathogen detection by multiple-labeling or label-free models such as fluorescence detection and surface plasmon resonance, electrochemical biosensors and lateral chromatography test strips, and their applications in pathogen detection and biomolecular screening. While notable progress has been made in the field in the last decade, challenges or drawbacks in their applications such as pathogen detection and biomolecular screening remain to be overcome.

## Introduction

Bacteria are microorganisms that are a few micrometers in length and morphologically described as rod, sphere or spiral. They can sense and respond to temperature and pH changes, nutritional starvation or new food sources, toxins, stresses, and quorum sensing signals (Salis et al., 2009). Pathogens are harmful species that cause infections and contagious diseases that result in many serious complications. Common bacterial pathogens and their complications include *Escherichia coli* and *Salmonella* (food poisoning), *Helicobacter pylori* (gastritis and ulcers), *Neisseria gonorrhoeae* (sexually transmitted disease), *N. meningitides* (meningitis), *Staphylococcus aureus* (boils, cellulitis, abscesses, wound infections, toxic shock syndromes, pneumonia, and food poisoning), and *Streptococcus* spp. (pneumonia, meningitis, ear infections, and pharyngitis). Worldwide, infectious diseases account for nearly 40% of the estimated total 50 million deaths annually (Ivnitski et al., 1999).

Detection and identification of microbial pathogens are crucial for public health and food safety (Law et al., 2015). Areas where detection of microbial pathogens is critical include clinical diagnosis, water and environmental analysis, food safety and biodefense. Currently, microbial culture-based tests and molecular assays (immunological or nucleic acid technologies) are among the most commonly used methodologies in detection and identification of microbial pathogens (Torres-Chavolla and Alocilja, 2009).

Aptamers are single stranded DNA or RNA ligands that can be selected for different targets starting from a huge library of molecules containing randomly created sequences (Tombelli et al., 2005); and these specifically selected nucleic acid sequences can bind to a wide range of non-nucleic acid targets with high affinity and specificity (Jayasena, 1999). Aptamers usually vary in length from 25 to 90 bases, and their typical structural motifs can be classified into stems (Tok and Cho, 2000), internal loops, purine-rich bulges, hairpin structures, hairpins, pseudoknots (Tuerk et al., 1992), kissing complexes (Boiziau et al., 1999), or G-quadruplex structures (Bock et al., 1992). The unique characteristics of aptamers such as their highly specific binding affinity to non-nucleic acid targets offer great potentials in the development of fast and efficient point-of-care assays for pathogen detection (Jayasena, 1999).

The selection process of aptamers is called systematic evolution of ligands by exponential enrichment (SELEX), which was developed by two independent groups in 1990 (Ellington and Szostak, 1990; Tuerk and Gold, 1990). Such work laid out the foundation for later developments of aptamers and aptamer-based technologies. Since then, SELEX has become a vital tool in selection of aptamers, transforming the great potential of aptamers and their related technologies in pathogen detection and biomolecular screening to a reality.

## Selection of Aptamers Against Bacterial Pathogens

### Conventional SELEX

Aptamers is evolved via an iterative process of SELEX (Hamula et al., 2006). The methodology consists screening large random oligonucleotide libraries through iterative cycles of *in vitro* selection and enzymatic amplification (Ellington and Szostak, 1990; Tuerk and Gold, 1990). Briefly, the selection consists of numerous cycles, and each cycle includes three steps: (i) an *in vitro* synthesized DNA or RNA library is incubated with the target; (ii) the target-bound and unbound nucleic-acid sequences are separated and the sequences that are not bound to the target are removed; and (iii) the target-bound sequences are used as the template for the subsequent PCR amplification. The selected sequences are used as the inputs in the next round of selection; and such selection cycle will continue until the desired sequence purity is achieved. In general, a random oligonucleotide library contains 40–100 single-stranded nucleotide sequences with a randomized stretch of nucleotide in the center and fixed sequences on each end. As many as 20 rounds of selection are carried out until a pool of aptamer sequences with high target affinity is obtained. These aptamers can then be cloned and sequenced (Hamula et al., 2006). After SELEX technology was established, a variety of aptamer-based methodologies have been developed for pathogen detection and biomolecular screening.

**FIGURE 1:**
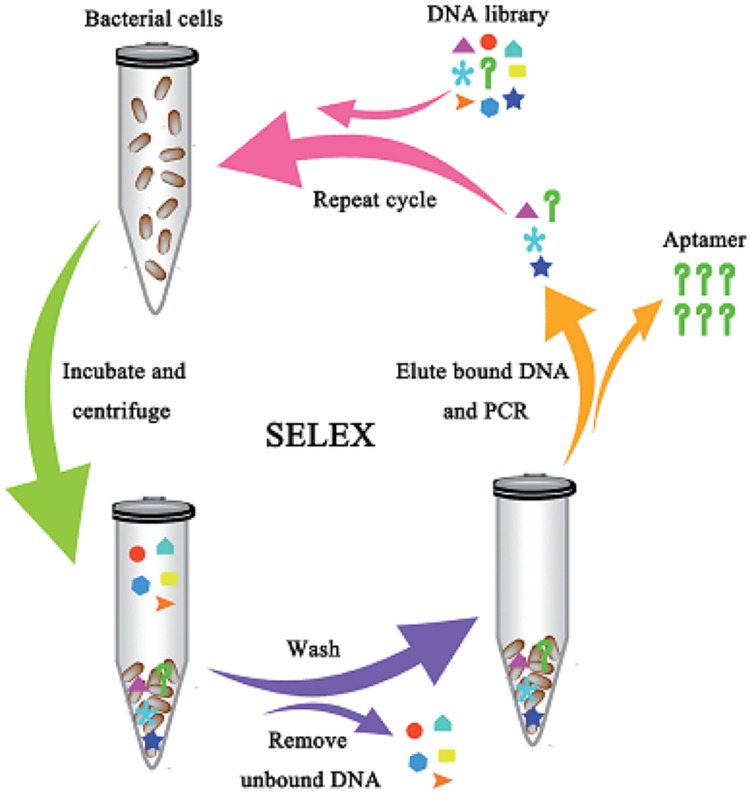
**Schematic showing the aptamer selection against live bacterial cells using whole-cell SELEX (Zhang et al., 2015)**.

**FIGURE 2:**
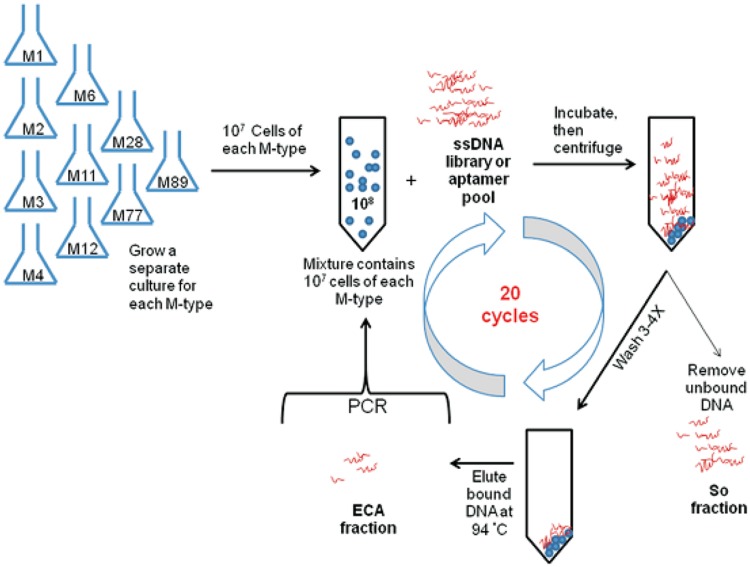
**Schematic of bacterial cell SELEX against a mixture of the 10 most prevalent GAS M-types in Canada (Liu G.Q. et al., 2014)**.

Most of the aptamers selected against pathogenic bacteria have been evolved using the conventional SELEX procedures as demonstrated in **Figure 1** (Zhang et al., 2015). Zhang et al. (2015) described the selection of DNA aptamers targeted *E. coli*. Two high-affinity aptamers to *E. coli* was obtained with totally eight rounds of SELEX selections. Furthermore, these conventional SELEX procedures have been used in the detection of various pathogens such as *Vibrio parahaemolyticus* (Hamula et al., 2011), *Salmonella typhimurium* (Duan et al., 2012), *Listeria monocytogenes* (Duan et al., 2013). These aptamers to a single bacterial species can be created within 10 rounds of selection, while those to multiple bacterial species also can be achieved within 20 rounds of selection as shown by the aptamer selection scheme in **Figure 2** (Liu G.Q. et al., 2014). As a result, Liu G.Q. et al. (2014) achieved selection of aptamers against various M-types of *S. pyogenes* by using these SELEX procedures rather than the conventional aptamer selection procedures, which use purified molecules of monoclonal cells as targets. It turned out that the aptamers selected through these procedures demonstrated high affinity and specificity to the targets (Liu G.Q. et al., 2014).

## Other Types of Selexs

### Whole-Cell SELEX

In addition to the conventional SELEX method, several novel SELEX methods have been developed. For example, a series of studies aimed to shorten the selection rounds in the aptamer manufacturing process. As a result, various methodologies with single round aptamer selection procedure have been developed, e.g., the ASExp (Aptamer Selection Express) method, which uses the magnetic mechanism for separation with microbeads (Fan et al., 2008).

A method called artificially expanded genetic information systems-SELEX (AEGIS-SELEX) was introduced in 2014 (Sefah et al., 2014). As the name suggests, this method uses artificially expanded genetic information systems for aptamer selection. An AEGIS-SELEX is started with a GACTZP DNA library, consisting of randomized sequences, primer sites and two modified nucleotides (ZP); and then a standard protocol for whole-cell SELEX is followed for the selection cycle. After the 12th selection round, the aptamers are sequenced and the aptamers’ affinity is evaluated. As a result, the AEGIS-SELEX method empowered the system with higher binding variations. The sequential aptamers can reach the nanomolar range and are expected to achieve higher sequence diversities nearer to that displayed by proteins (Sefah et al., 2014).

### Genomic SELEX

**FIGURE 3:**
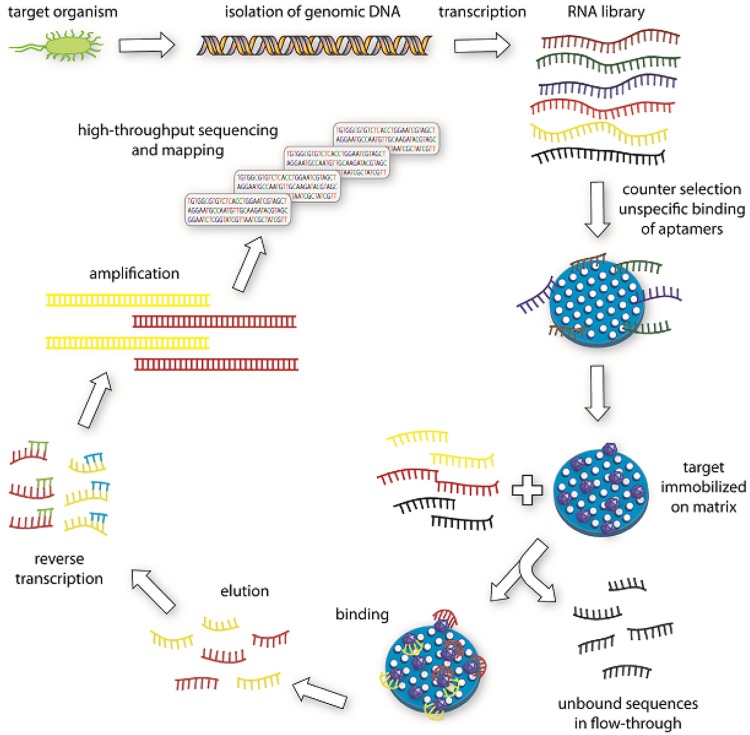
**Illustration of genomic SELEX (Lorenz et al., 2010).** Genomic DNA is isolated from the target organism.

Apart from whole-cell SELEX, SELEX can be generated in the nucleic acid level as well. Lorenz et al. (2006) introduced genomic SELEX. A genomic DNA library is used in genomic SELEX in contrast to a conventional SELEX, in which a chemically synthesized library is used (Lorenz et al., 2006, 2010). A final pool of oligonucleotides obtained from the ninth selection round is characterized by high throughput sequencing (HTS) as shown in **Figure 3** (Lorenz et al., 2010). A distinctive advantage of genomic SELEX is the dramatic decrease of the diversity of the initial library. Genomic DNA is isolated from the target organism and the initial library is prepared by adding specific primers to the isolated DNA and followed by Klenow-fragment extension on the new strand. This initial library is transcribed into RNA, and then continued for the selection process. First, a counter selection against immobilization matrix step is performed. The library sequences are incubated with the target and the bound oligonucleotides are subjected to reverse transcription into cDNA and used in the next round of selection. After several more rounds, the enriched sequences are subjected to HTS and mapping analyses as shown in **Figure 3** (Lorenz et al., 2010).

Additionally, a method called transcriptomic SELEX, which is similar to genomic SELEX, has been developed (Fujimoto et al., 2012), whereas genomic SELEX opened up a new horizon for the determination of functionally active motifs in genomic DNA. One disadvantage of this method was the non-specific binding in the assay, i.e., primer binding regions in a DNA library could easily anneal with some random fragments in the genomic library. Afterward, Jin-Der and Gray (2004), developed primer-free genomic SELEX to remedy the drawbacks associated with the transcriptomic SELEX.

## Applications in Pathogen Detection and Biomolecular Screening

Currently, aptamer-based detection methods can be used in public health and food safety are limited. A primary reason for that might be the complexity of the methods since these methods involve a variety of techniques in the sample preparation and detection processes such as sample’s extraction, purification, enrichment, and separation (Pitcher and Fry, 2000; Stevens and Jaykus, 2004).

Pathogen detection is important for public health and food safety. Three areas of application account for over two thirds of all research in the field of pathogen detection (Lazcka et al., 2007), including the food industry (Patel, 2002; Leonard et al., 2003), water and environment quality control (Emde et al., 1992; Theron et al., 2000), and clinical diagnosis (Atlas, 1999). The remaining efforts go into fundamental studies (Herpers et al., 2003; Gao et al., 2004), method performance studies (Dominguez et al., 1997; Taylor et al., 2005), and development of new applied methods (Ko and Grant, 2003; Yoon et al., 2003).

Aptamers and antibodies are commonly used reagents in various detection assays; the affinity of aptamers to their targets is comparable to, or even higher than most of the monoclonal antibodies to their targets; typical dissociation constants of aptamer-target complexes are found to be in the picomolar to low micromolar ranges (Hamula et al., 2006). Therefore, nucleic-acid aptamers demonstrate numerous advantages as recognition elements in biosensing over the traditional antibodies. Moreover, aptamers are small in size, chemically stable and cost effective. More importantly, aptamers provide remarkable flexibility and convenience in engineering their structures, which have led to the development of novel biosensors that exhibited high sensitivity and specificity (Song et al., 2008). In addition to these above-mentioned advantages, aptamers offer some distinctive characteristics as a biological reagent, i.e., once selected, it can be synthesized with high reproducibility and purity from commercial sources. Furthermore, in contrast to protein-based antibodies or enzymes, aptamers (made of DNA) usually are chemically stable and often undergo significant conformational changes upon target binding (Song et al., 2008; Binnin et al., 2011). Nucleic acid aptamers are widely used in the field of biosensors. Therefore, numerous aptamer-based biosensors are developed to detect bacterial pathogens. Hence, in this review, we also summarize the most commonly used aptamer-based biosensors and bioassay methods for detection of bacterial pathogens.

## Optical Biosensors

Optical biosensors are probably the most popular in bioanalysis, due to their selectivity and sensitivity. Optical biosensors have been developed for rapid detection of contaminants (Willardson et al., 1998; Tschmelak et al., 2004), toxins, drugs (Bae et al., 2004), and bacterial pathogens (Baeumner et al., 2003).

## Label-Free Detection of Bacteria

Several techniques have been described that allow direct, label-free monitoring of cells at solid-liquid interfaces (Ebato et al., 1994; Morgan et al., 1996; Piehler et al., 1996; Medina et al., 1997; Ghindilis et al., 1998; Fratamico et al., 1998). These techniques are based on direct measurement of a physical phenomenon occurring during the biochemical reactions on a transducer surface (Ivnitski et al., 1999); changes in pH, oxygen consumption, potential difference, current, resistance, ion concentrations, and optical properties can be used as measures of signal parameters in certain detection systems.

## Fluorescence Detection

**FIGURE 4:**
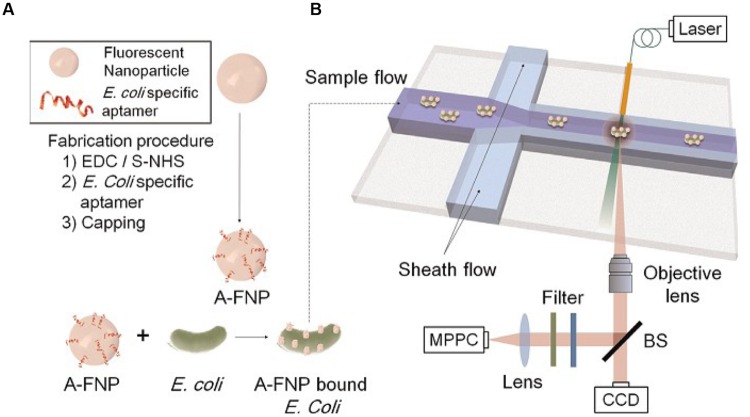
**Schematics of the present microbe detection system. (A)** Preparation of aptamer conjugated fluorescence nanoparticles (A-FNPs). **(B)** Detection of A-FNP-bound *E. coli* by the microchannel and optical particle counter.

Fluorescence occurs when a valence electron is excited from its ground state to an excited singlet state. The excitation is produced by the absorption of light of sufficient energy. When the electron returns to its original ground state it emits a photon at lower energy (Chung et al., 2014). Nowadays, the fluorescent material is also broadly used (Vigneshvar et al., 2015). Bacteria can be detected by using fluorescent beads as shown in **Figure 4** (Chung et al., 2014). Fratamico et al. (1998) developed a real-time, continuous, and non-destructive single cell detection method that uses target specific aptamer-conjugated fluorescent nanoparticles (A-FNPs) and an optofluidic particle-sensor platform to detect bacterial pathogens.

## Surface Plasmon Resonance Based Detections

**FIGURE 5:**
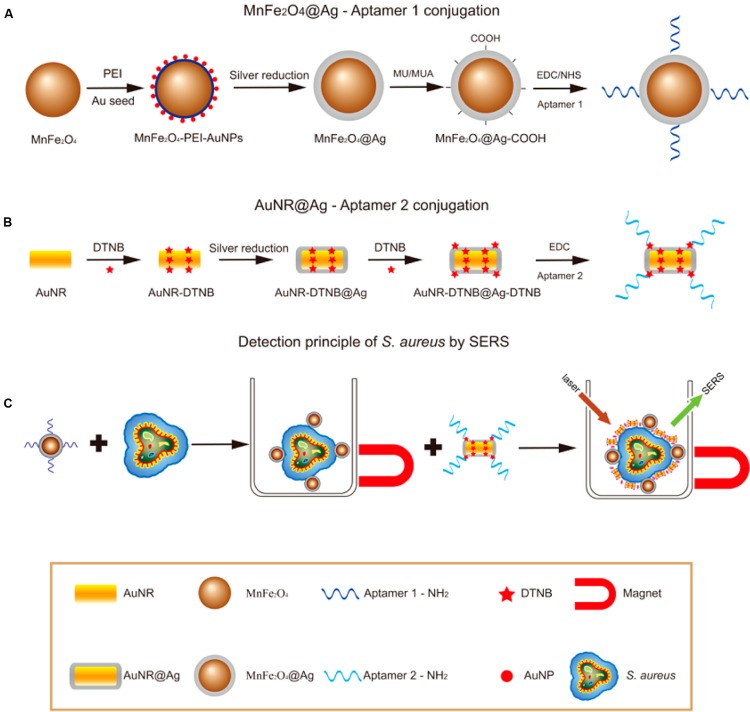
**Flowchart of *S. aureus* detection using SERS (Wang et al., 2015). (A)** Synthesis of monodispersed silver-coated magnetic nanoparticles and their conjugation with aptamer 1. **(B)** Synthesis of core-shell plasmonic nanoparticles (AuNR-DTNB@Ag-DTNB) and their conjugation with aptamer 2. **(C)** Schematic illustration of the operating principle for *S. aureus* detection.

Surface plasmon resonance (SPR) biosensors (Cooper, 2003) measure changes in refractive index caused by structural alterations in the vicinity of a thin film metal surface. Given its high sensitivity and fingerprinting capability, surface-enhanced Raman scattering (SERS) has been applied in various fields (Nie and Emory, 1999; Cao et al., 2002; Li et al., 2010). The detection and identification of pathogenic microorganisms by SERS have recently drowned attention because of the potential application of this technology in single-cell detection (Chang et al., 2013). The flow chart in **Figure 5** demonstrates the process of *S. aureus* detection by using SERS (Wang et al., 2015). A plan f or the conjunction of aptamers to Ag-MNPs is shown in **Figure 5A**; a novel SERS tag (DioPNPs) was designed as shown in **Figure 5B**; and the operating principle of the SERS biosensor for bacterial detection that is based on aptamer recognition is shown in **Figure 5C**.

## Electrochemical Biosensors

**FIGURE 6:**
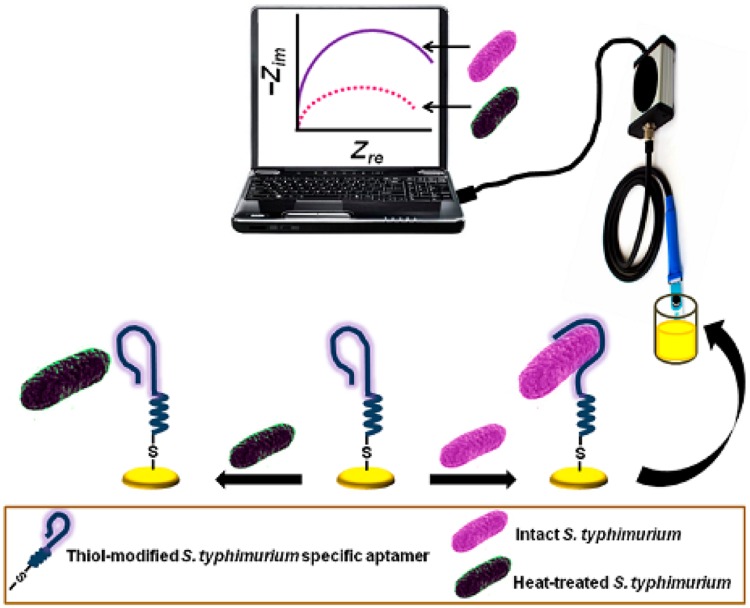
**Schematic diagram of the aptamer-mediated electrochemical detection of live *Salmonella* Typhimurium bacteria (Jenkins et al., 1988)**.

Electrochemical sensors have several advantages over optical-based systems in that they can operate in turbid media, offer comparable instrumental sensitivity, and are more amenable to miniaturization. Modern electroanalytical techniques can reach an extremely low limit of detection (up to 10^-9^ M), which can be achieved by using small volumes (1–20 mL) of samples (Mahmoud et al., 2012). A typical electrochemical method, which is illustrated in **Figure 6** (Jenkins et al., 1988), provides a brand new avenue for the aptamer-based viability detection of various microorganisms, particularly viable but non-cultural (VBNC) bacteria, using a rapid, economic, and label-free electrochemical platform as Jenkins et al. (1988) reported.

**FIGURE 7:**
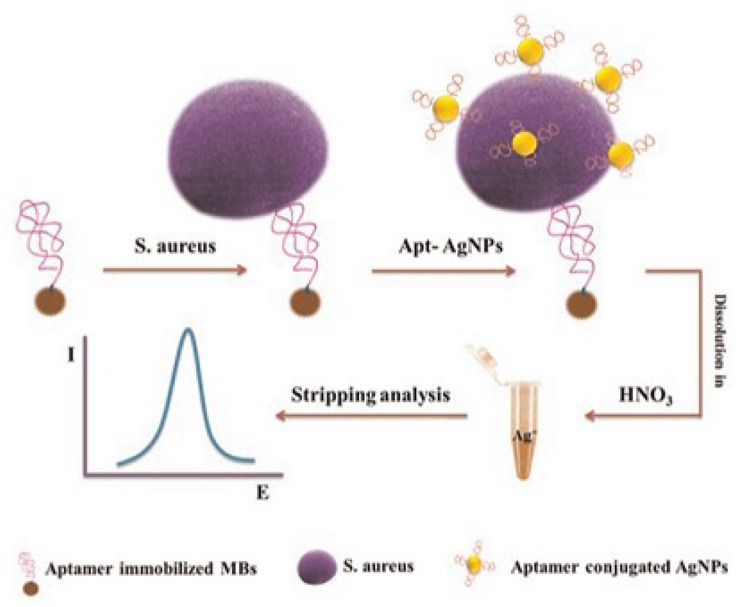
**Schematic representation of dual-aptamer-based electrochemical sandwich immunosensor for the detection of *S. aureus* (Abbaspour et al., 2015)**.

Recently, a newly developed aptamer-based biosensor system has been developed to detect pathogen (Abbaspour et al., 2015). This system uses a sensitive and highly selective dual-aptamer-based sandwich immunosensor in conjunction with electrochemical means for the detection of *S. aureus* as shown by **Figure 7** (Abbaspour et al., 2015). As a result, excellent discriminatory power of the biosensor was achieved by utilizing the two specific aptamer sequences against the target bacteria and the magnetic beads to capture *S. aureus* in a liquid phase. The electrochemical detection method demonstrated a few advantages in term of simplicity, turn-around time, low cost, and limit of detection compared to the conventional detection methods. The superior sensitivity of this method provides the possibility to use aptamers to detect extremely low number (in single digital) of pathogenic bacteria in foods, which is hardly achievable by the conventional methods. Also, researchers have tried to use biotinylated single-stranded (ss) DNA aptamers with *L. monocytogenes* binding specificity to capture the bacteria, and subsequently detected the organism in qPCR assay. Biotinylated ssDNA aptamers are promising ligands that can be used for concentrating foodborne pathogens prior to using the conventional molecular approaches for detection (Suh and Jaykus, 2013).

## Lateral Chromatography Test Strips

**FIGURE 8:**
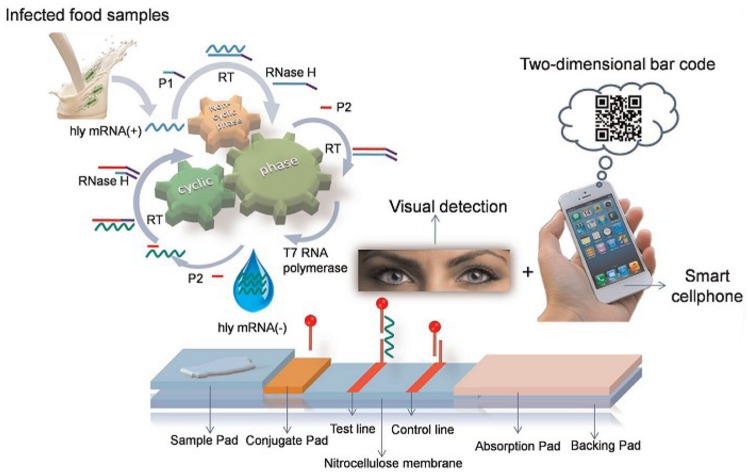
**Schematic illustration of isothermal RNA amplification and the configuration of the bioactive paper-based platform (Chung et al., 2014)**.

Lateral chromatography test strips, whose mechanism is illustrated in **Figure 8**, are also widely used. For example, Liu H.X. et al. (2014) demonstrated a simple and sensitive method for visual detection of viable pathogenic bacteria based on an isothermal RNA amplification reaction-based bioactive paper-based platform by using a two-dimensional barcode as the receiving/transmitting media for rapid detection.

Numerous assays based on the specific binding of an antibody to an antigen, such as enzyme-linked immunosorbent assay (ELISA) (Zunabovic et al., 2011; Hsu et al., 2014) and immunochromatographic lateral flow test strips (Ge et al., 2012; Nash et al., 2012; Yan et al., 2012; Cho and Irudayaraj, 2013), have been developed. However, rapid immune tests, which are widely used in low resource settings, are not suitable for fast foodborne pathogen detection due to their low sensitivity. Finally, a low-cost platform was constructed for viable pathogen detection with the naked eyes (Liu H.X. et al., 2014). In that system, specifically amplified products were applied to a paper-based platform to perform sandwich hybridization and followed by a visual exam. This method is suitable for point-of-care applications to detect foodborne pathogens (Liu H.X. et al., 2014).

## Conclusions and Perspectives

In this review, we summarize the most commonly used SELEX methods in selection of aptamers against bacterial foodborne pathogens and the application of aptamer-based biosensors in biomolecular screening. Although SELEX advanced rather slowly initially, the selection of aptamers against pathogenic bacteria has been stably progressing in the last decade and nowadays, this technology has been evolved into a useful tool in pathogen detection and biomolecular screening. Initially, conventional steps and PCR were used in the SELEX procedures in the early years and then, several novel approaches and new biological materials were adapted in the SELEX procedures. On the other hand, targeting bacterial cells for detection purpose by SELEX also encounters some drawbacks, because bacteria’s highly variable and complex structures may influence the performance of aptamers. Therefore, it is necessary to continue to develop simpler and more efficient SELEX methods (requiring fewer rounds in selection) to generate specific and/or universal aptamers against various bacterial pathogens.

Compared to traditional antibody generation process, SELEX can efficiently generate specific nucleic acid probes against various analytes in a relatively short period of time. More importantly, the extraordinary properties of the selected aptamers, such as easy scale-synthesis, easy modification and long-term stability, make aptamers ideal alternatives to the traditional antibodies. However, improvement in aptamer selection efficiency by SELEX is needed in the future work. Also, other platforms, including magnetic separation techniques, array or microfluidic chips, can be integrated with initial SELEX to further widen the applications of this promising technology. For example, at present, biosensor-based detection technologies can merely meet the basic requirements for testing in the laboratory and clinic. Obviously, it can be expected that simpler, faster, more efficient, and more economic aptamer-based methods will be developed for pathogen detection and biomolecular screening in the future.

## Author Contributions

JT, FY, LZ, YY, LY, FX, and BL wrote the manuscript. WC and BL revised the manuscript.

## Conflict of Interest Statement

The authors declare that the research was conducted in the absence of any commercial or financial relationships that could be construed as a potential conflict of interest.
